# Manganese-52: applications in cell radiolabelling and liposomal nanomedicine PET imaging using oxine (8-hydroxyquinoline) as an ionophore†
†Electronic supplementary information (ESI) available. See DOI: 10.1039/c8dt00100f. The data supporting this research are openly available from the King's College London research data archive at http://dx.doi.org/10.18742/RDM01-283.


**DOI:** 10.1039/c8dt00100f

**Published:** 2018-05-24

**Authors:** Peter Gawne, Francis Man, Jesper Fonslet, Riya Radia, Jayanta Bordoloi, Matthew Cleveland, Pilar Jimenez-Royo, Alberto Gabizon, Philip J. Blower, Nicholas Long, Rafael T. M. de Rosales

**Affiliations:** a School of Biomedical Engineering & Imaging Sciences , King's College London , St Thomas’ Hospital , London , SE1 7EH , UK . Email: rafael.torres@kcl.ac.uk; b The Hevesy Lab , Technical University of Denmark , 4000 Roskilde , Denmark; c GSK Medicines Research Centre , Gunnels Wood Road , Stevenage , Hertfordshire , SG1 2NY , UK; d Oncology Institute , Shaare Zedek Medical Center and Hebrew University-School of Medicine , Jerusalem 9103102 , Israel; e Department of Chemistry , Imperial College London , South Kensington Campus , London SW7 2AZ , UK

## Abstract

The ionophore 8-hydroxyquinoline (oxine) has been used to radiolabel cells and liposomal medicines with ^111^In and, more recently, ^89^Zr, for medical nuclear imaging applications. Oxine has also shown promising ionophore activity for the positron-emitting radionuclide ^52^Mn that should allow imaging of labelled cells and nanomedicines for long periods of time (>14 days). However, to date, the radiometal complex formed and its full labelling capabilities have not been fully characterised. Here, we provide supporting evidence of the formation of [^52^Mn]Mn(oxinate)_2_ as the metastable complex responsible for its ionophore activity. The cell labelling properties of [^52^Mn]Mn(oxinate)_2_ were investigated with various cell lines. The liposomal nanomedicine, DOXIL® (Caelyx) was also labelled with [^52^Mn]Mn(oxinate)_2_ and imaged *in vivo* using PET imaging. [^52^Mn]Mn(oxinate)_2_ was able to label various cell lines with moderate efficiency (15–53%), however low cellular retention of ^52^Mn (21–25% after 24 h) was observed which was shown not to be due to cell death. PET imaging of [^52^Mn]Mn-DOXIL at 1 h and 24 h post-injection showed the expected pharmacokinetics and biodistribution of this stealth liposome, but at 72 h post-injection showed a profile matching that of free ^52^Mn, consistent with drug release. We conclude that oxine is an effective ionophore for ^52^Mn, but high cellular efflux of the isotope limits its use for prolonged cell tracking. [^52^Mn]Mn(oxinate)_2_ is effective for labelling and tracking DOXIL *in vivo*. The release of free radionuclide after liposome extravasation could provide a non-invasive method to monitor drug release *in vivo*.

## Abstract 2

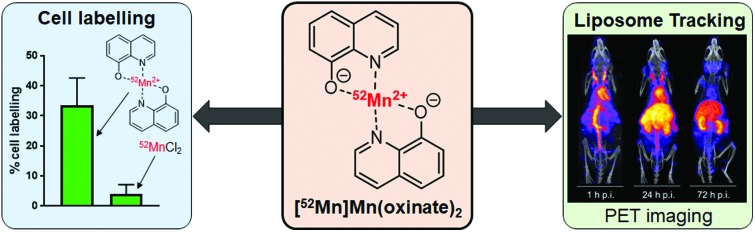
The ionophore 8-hydroxyquinoline (oxine) has been used to radiolabel cells and liposomal nanomedicines with the PET radiometal manganese-52.

## Introduction

**Scheme 1:**
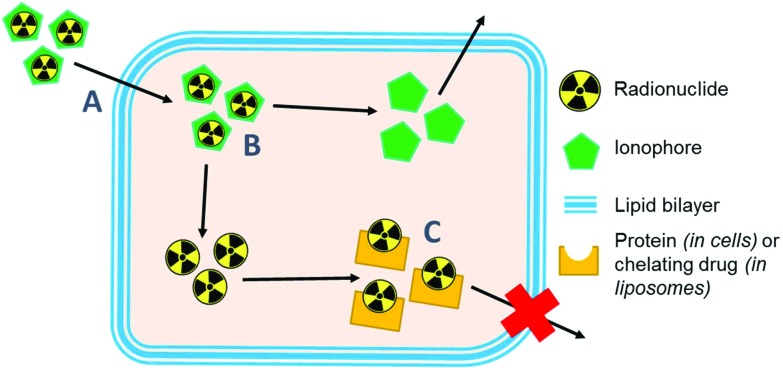
Diagram showing the proposed mechanism of labelling cells and liposomes using radio-ionophore complexes. (A) The neutral lipophilic radio-ionophore complex crosses lipid bilayer. (B) The meta-stable complex dissociates and (C) the radio-metal binds to intracellular proteins/macromolecules or drugs with chelating groups within liposomal medicines.

Ionophores are organic ligands that facilitate transport of metal ions passively across lipid bilayers. In particular, ionophores have been widely used since the 1970s to directly label and image both cells and liposomal medicines *in vivo* with radiometals using clinical nuclear imaging techniques such as single-photon emission tomography (SPECT) and more recently positron emission tomography (PET). Ionophore ligands are usually lipophilic and have low denticity. Binding of the radiometal with the ionophore results in a complex that is both lipophilic and uncharged, and able to passively cross lipid bilayers (Scheme 1). The radiometal-ionophore complexes are commonly meta-stable and dissociate inside the cell/liposome, at which point trapping occurs *via* the binding of the radiometal to intracellular proteins^1^ or intraliposomal drug molecules – provided they have chelating groups – or other metal-chelating ligands (Scheme 1).^2^ As such, effective radio-ionophore agents should facilitate fast uptake and slow radionuclide efflux, whilst not affecting the viability or function of cells/liposomes.

**Fig. 1:**
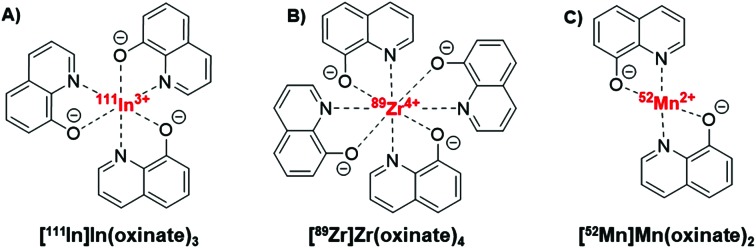
Structures of the radio-ionophore complexes discussed: [^111^In]In(oxinate)_3_ (A), [^89^Zr]Zr(oxinate)_4_ (B) and [^52^Mn]Mn(oxinate)_2_ (C).

The longitudinal imaging/tracking of living cells and liposomal nanomedicines within a living organism has applications in locating inflammation (labelled leukocytes) and determining the biodistribution of therapeutic cells and nanomedicines. To allow this, the choice of radionuclide is important. One of the most widely used radio-ionophore complexes to date is the tris(oxinate) complex of the gamma-emitting radionuclide ^111^In (*t*_1/2_ = 2.8 days) (Fig. 1A). Known as “[^111^In]In-oxine” this compound has been used clinically since the 1980s to radiolabel autologous leukocytes for imaging of infection and inflammation *in vivo*,^3^,^4^ and was recently withdrawn from the EU market due to the perceived low cost-effectiveness by the supplier.^5^ More recently, it has regained attention for its ability to directly label and track a variety of immune cells.^6^–^11^ With the growing availability of PET, focus has shifted towards the development of PET tracers for cell labelling. PET imaging offers higher sensitivity than SPECT imaging as well as improved spatial resolution and quantification in the clinical setting.^12^ In particular, ^89^Zr complexes (*t*_1/2_ = 3.27 days; *β*^+^ = 22.3%) have been investigated as PET alternatives to [^111^In]In-oxine. Zirconium and indium have similar preferred ligand types – despite the different preferred oxidation states (In(iii) and Zr(iv)) – as well as having similar half-lives,^1^ and the PET tracer [^89^Zr]Zr(oxinate)_4_ (also known as [^89^Zr]Zr-oxine, Fig. 1B) has shown excellent cell labelling and tracking properties.^1^,^13^ The longer half-life of ^89^Zr, combined with improved cellular retention of the radionuclide compared to ^111^In,^13^ allowed more prolonged *in vivo* cell tracking with PET (7–14 days) with various cell types.^13^–^15^ [^89^Zr]Zr(oxinate)_4_ has also been used to directly label and track liposomal medicines *in vivo* for up to 7 days, without the need for modification of the nanomedicine or interference with its manufacture.^2^

In our search for new radiometals to track cells/nanomedicines with PET for longer periods of time we turned our attention towards ^52^Mn (*t*_1/2_ = 5.59 days, *β*^+^ = 29.6%). Recently we have shown that oxine has promising ^52^Mn ionophore activity *in vitro* using liposomes as a model.^2^ The radiolabelling yields and *in vitro* serum stability properties where comparable to those obtained with [^89^Zr]Zr(oxinate)_4_. However, the identity of the [^52^Mn]Mn-oxine complex was not known and its cell labelling and *in vivo* liposome tracking ability was unexplored. Here, we describe the synthesis and characterisation of the radiometal complex [^52^Mn]Mn(oxinate)_2_ (Fig. 1C) and evaluated its cell-labelling properties. Additionally, the *in vivo* stability and biodistribution of ^52^Mn-labelled liposomes, radiolabelled with this radiotracer, were investigated in mice with PET imaging using the clinically approved nanomedicine DOXIL® (Caelyx).

## Results and discussion

### Radiosynthesis of [^52^Mn]Mn(oxinate)_2_

**Fig. 2:**
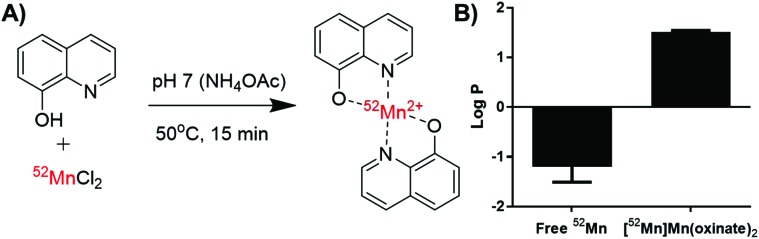
(A) Radiochemical scheme for the synthesis of [^52^Mn]Mn(oxinate)_2_, and (B) chart showing the log *P* values of unchelated ^52^Mn and [^52^Mn]Mn(oxinate)_2_ in water/octanol. Error bars represent standard deviation (*n* = 2).

[^52^Mn]Mn(oxinate)_2_ can be synthesised rapidly and reliably by the addition of oxine (from a DMSO stock solution) to [^52^Mn]MnCl_2_ in dilute HCl, followed by neutralisation with 0.1 M ammonium acetate solution (pH 7) and a brief heating step at 50 °C (Fig. 2A). Instant thin layer radiochromatography (iTLC) analysis using a mobile phase of 25% methanol in chloroform shows that whereas [^52^Mn]MnCl_2_ stays at the baseline (*R*_f_ = 0) radioactivity of the product solution migrates with the solvent, indicating the formation of the expected lipophilic compound. *R*_f_ values for the oxine compound were inconsistent (*R*_f_ = 0.3–0.8) most likely due to varying amounts of DMSO present in the reaction mixture. The radiochemical yield (RCY) was 69 ± 20% (*n* = 3) based on iTLC analysis, which we also used as an estimate of the radiochemical purity. The lipophilicity of [^52^Mn]Mn(oxinate)_2_ was confirmed with log *P* measurements using octanol/water solvent extraction (log *P*_water_ = 1.5 ± 0.1), whereas the log *P* of ^52^MnCl_2_ showed the expected high hydrophilicity of a hydrated manganese ion (log *P* = –1.2 ± 0.3) (Fig. 2B). The synthesis of [^52^Mn]Mn(oxinate)_2_ has benefits over [^89^Zr]Zr(oxinate)_4_: it does not require the solvent extraction step required to remove oxalate/oxalic acid from the final [^89^Zr]Zr(oxinate)_4_ product, involving vigorous vortexing followed by separation and evaporation of the organic layer (CHCl_3_). Sato *et al.* recently reported an improved synthetic method for [^89^Zr]Zr(oxinate)_4_ from [^89^Zr]ZrCl_4_ in aqueous media, however vortexing of the mixture was still necessary.^14^

### Synthesis and characterisation of ^nat^Mn(oxinate)_2_

To determine the chemical identity of the species formed during radiosynthesis we synthesised and characterised the non-radioactive ^55^Mn(oxinate)_2_ complex (^55^Mn = ^nat^Mn = Mn = naturally occurring Mn). This was achieved by addition of 0.5 equivalents of MnCl_2_ to a basic solution of 8-hydroxyquinoline – deprotonated with one equivalent of potassium hydroxide – resulting in the formation of a pale yellow precipitate. Formation of the bis-oxinate Mn(ii) complex was confirmed with mass spectrometry (electrospray in positive mode) with a *m*/*z* peak at 344.0348 consistent with a protonated Mn(oxinate)_2_ complex (M + 1) and another peak at 687.0684 relating to a protonated 2[Mn(oxinate)_2_] species (2M + 1) (Fig. S1†). No peaks were observed with *m*/*z* matching a ligand-to-metal stoichiometric ratio of 3 : 1, suggesting that the oxidation of the metal to form a Mn(oxinate)_3_ complex had not occurred. Additionally, IR spectroscopy showed a reduction of the broad O–H stretch band at 2700–3400 cm^–1^ upon metal complexation – relating to the deprotonation of the hydroxyl associated with formation of the Mn–O bond (Fig. S2†). Further analysis of Mn(oxinate)_2_ complex was limited by its insolubility in all the organic solvents tested. Whilst previous work has reported the synthesis of Mn(oxinate)_2_,^16^,^17^ few reports have characterised the structure of the manganese complex. Li *et al.* reported the 1D coordination polymer of [Mn(oxinate)_2_]_n_ and showed each Mn^2+^ ion was bound to the oxine ligand in a severely distorted N_2_O_4_ octahedral geometry.^18^ However the high temperature (453 K) synthesis method used in this case limits comparability with the compound synthesised herein.

**Fig. 3:**
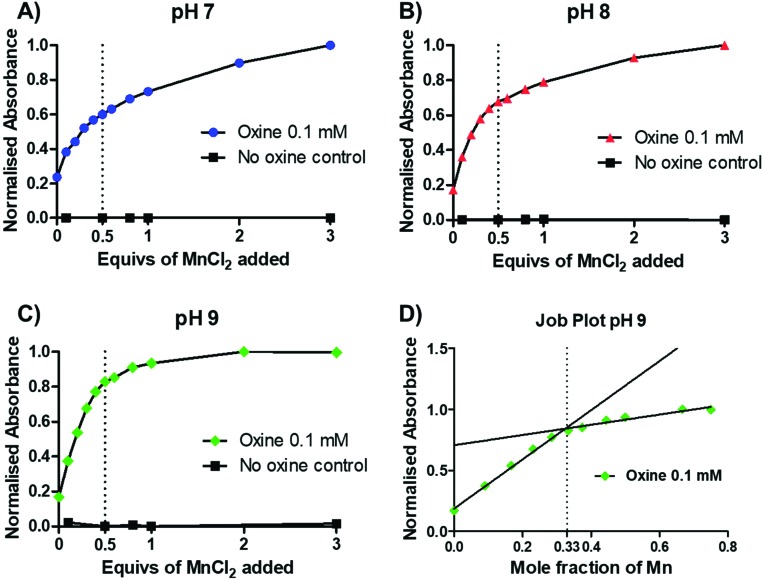
UV-vis titration (*λ* = 255 nm) of MnCl_2_ into a 0.1 mM solution of oxine (1.46% v/v DMSO in 1 mM ammonium acetate solution) performed at room temperature at (A) pH 7; (B) pH 8; (C) pH 9; (D) a Job plot of mole fraction (fraction of reagent in solution) of MnCl_2_*versus* absorbance. Mole fraction of Mn = [*χ*_Mn_/(*χ*_Mn_ + *χ*_oxine_)] where *χ*_Mn_ = total moles of MnCl_2_ in solution and *χ*_oxine_ = total moles of oxine in solution.

To gain further insight into the preferential stoichiometry of the complex being formed in the radiochemical reaction, UV-vis spectrophotometric titrations were carried out (representative spectra in Fig. S3†). Unlike other characterisation spectroscopic methods such as NMR, the low concentrations of reactants required for UV-vis spectrometry are comparable to those in radiochemistry which is always performed at low radiometal concentrations and metal-to-ligand ratios. Thus, the change in the absorbance spectrum of a 0.1 mM 8-hydroxyquinoline solution (1.46% v/v DMSO in 1 mM ammonium acetate solution) was monitored upon titration of increasing equivalents of MnCl_2_ at pH 7–9 (Fig. 3A–C and S3†). Upon addition of MnCl_2_, an increase in an absorption band with *λ*_max_ = 255 nm was observed across each pH. In the absence of oxine, no increase in absorbance was seen, demonstrating that the absorbance increase is due to metal–ligand binding. At each pH, a decrease in the absorbance change occurred after 0.5 equivalents of MnCl_2_, indicating the preferential formation of the bis-oxine complex. This was particularly prominent with increasing pH values (*e.g.* pH 9) which is consistent with the relatively high p*K*_a_ of the hydroxyl group from the oxine ligand (p*K*_a_ = 11.54).^19^ Additionally, the absorbance at 255 nm was plotted as a function of the mole fraction (relative proportion, or fraction, of a compound in solution) of MnCl_2_ added (known as a Job plot^20^). The Job plot in Fig. 3D showed that the change in absorption decreased once a mole fraction of MnCl_2_ reached 0.33, characteristic of a ML_2_ complex (where M = Mn; L = 8-hydroxyquinoline). In the case of Mn(oxinate)_3_ (ML_3_) being preferentially formed, the absorption would be seen to decrease at a mole fraction of 0.25 – which we did not observe. Additionally, electron paramagnetic resonance (EPR) spectroscopy of a solution of 1 mM oxine (1.46% v/v DMSO in 1 mM ammonium acetate solution) mixed with 0.5 equivalents of MnCl_2_ showed the presence of Mn^2+^ (Fig. S4†). These results lead us to propose that the chemical species formed during the radiosynthesis is [^52^Mn]Mn(oxinate)_2_.

Another important aspect of the direct cell radiolabelling strategy is that the compound (*i.e.* Mn(oxinate)_2_) must be metastable, with sufficient stability to cross the cell membrane but unstable enough to decompose inside the cell releasing the radiometal (Scheme 1). To test the metastability of Mn(oxinate)_2_, we monitored its absorbance at 255 nm using the same conditions as in the UV-vis titrations. We observed gradual decomposition of the complex indicated by a decrease in the absorption (Fig. S5†). This process, however, is slow and only a small fraction of decomposition is evident after 1 h. Taking into account that direct cell labelling using ionophores is fast (<30 min), and that metastability is required for metal release inside the cells, these results further support the use of this complex for direct cell labelling.

### *In vitro* cell labelling with [^52^Mn]Mn(oxinate)_2_

**Fig. 4:**
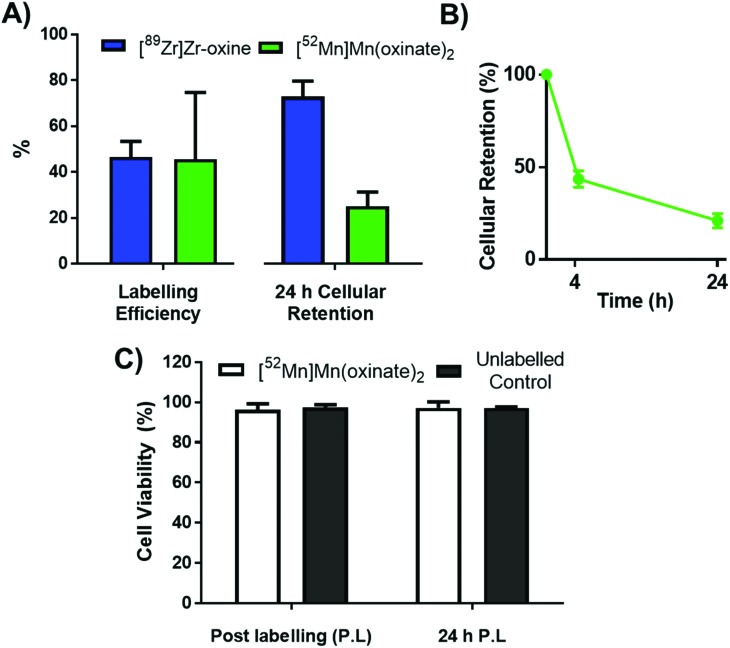
(A) Graph comparing the labelling efficiency (left) and cellular retention after 24 h (right) of [^52^Mn]Mn(oxinate)_2_ (green) with that of [^89^Zr]Zr(oxinate)_4_ (blue) in gamma-delta (γδ) T-cells. (B) Cellular retention of ^52^Mn over 24 h in MDA-MB 231 cells. (C) Cell viability of MDA-MB 231 cells radiolabelled with [^52^Mn]Mn(oxinate)_2_ (50 KBq per 10^6^ cells) compared to unlabelled controls. All error bars represent standard deviation (*n* = 3).

**Fig. 5:**
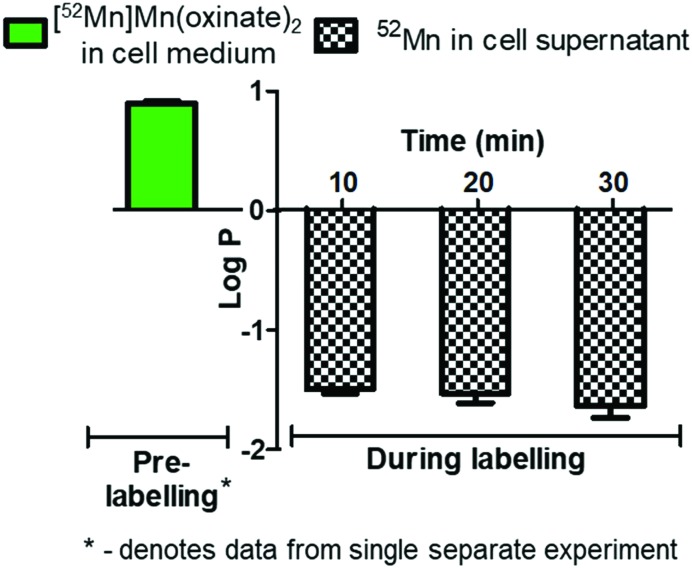
Log *P* analysis of cell supernatant of MDA-MB 231 cells labelled with [^52^Mn]Mn(oxinate)_2_. Log *P* values of supernatant taken during the 30 min labelling period show a negative (hydrophilic value) compared with the lipophilicity value of [^52^Mn]Mn(oxinate)_2_ in cell-free medium (green bar on left). Error bars represent standard deviation (*n* = 3) except for the log *P* of [^52^Mn]Mn(oxinate)_2_ which is a single experiment performed in triplicate.

The cell labelling properties of [^52^Mn]Mn(oxinate)_2_ were assessed in three different cell lines and in human platelets. Table 1 summarises the cell labelling efficiencies and cellular retention of ^52^Mn. Across all cell types, the presence of oxine resulted in higher cellular uptake compared to that achieved with the unchelated ^52^Mn control, demonstrating the ionophore properties of oxine for Mn(ii). The presence of excess amounts of a similar divalent metal such as Ca^2+^ in the cell medium (1.8 mM) during the MDA-MD-231 radiolabeling experiments, compared to the pM-nM concentrations of [^52^Mn]Mn(oxinate)_2_, demonstrates sufficient complex inertness for direct cell labelling. This was surprising due to the similarity of these two metals and the expected lability of the Mn(oxinate)_2_ complex. Furthermore, UV-vis studies demonstrated oxine selectivity for Mn^2+^ by showing complete formation of ^nat^Mn(oxinate)_2_ even in the presence of a large excess (up to 20 eq.) of Ca^2+^ (Fig. S6†). The cell labelling ability of [^52^Mn]Mn(oxinate)_2_ was directly compared to that of [^89^Zr]Zr(oxinate)_4_ in gamma-delta T-cells. Interestingly, [^52^Mn]Mn(oxinate)_2_ showed comparable cell labelling efficiency to [^89^Zr]Zr(oxinate)_4_ (45.6 ± 29.1% and 46.6 ± 6.8%, respectively), suggesting that the number of oxine ligands in the primary coordination sphere of these metal complexes is not an important factor. Retention of ^52^Mn in these cells after 24 h, however, was approximately three times lower than that of ^89^Zr (27.1 ± 6.8% and 74.9 ± 6.2%, respectively) (Fig. 4A). Further analysis with ^52^Mn-labelled MDA-MB-231 breast cancer cells showed that cellular efflux of ^52^Mn from labelled cells occurs rapidly with over 50% of the initial intracellular ^52^Mn leaving the cells after 4 hours. This result confirms this efflux is fast and not cell-specific, at least for the cells tested in this work (Fig. 4B). A possible explanation for this rapid efflux of the radiometal is cell death due to the radiolabelling process. However, a cell viability assay comparison (trypan blue) showed no different between MDA-MB-231 cells labelled with [^52^Mn]Mn(oxinate)_2_ (0.05 Bq per cell) and non-labelled cells, both immediately and 24 h after labelling (Fig. 4C). To investigate the cellular efflux of ^52^Mn further, aliquots of the cell supernatant were taken at various time points during the 30 min labelling period of MDA-MB-231 cells and lipophilicity measurements of this solution were performed to identify the nature of the radioactive species. At each time point log *P* values of the ^52^Mn species in the supernatant were negative/hydrophilic (*ca.* –1.5) whereas the log *P* value for [^52^Mn]Mn(oxinate)_2_ in cell-free medium was positive/lipophilic (0.88) (Fig. 5). These results indicate that: (i) ^52^Mn, after entering the cell as part of a lipophilic compound, leaves the cell in a hydrophilic form, and (ii) the hydrophilic log *P* values obtained during incubation cannot be fully explained by decomposition of [^52^Mn]Mn(oxinate)_2_ in cell medium.

**Table 1:** Cell labelling efficiency of [^52^Mn]Mn(oxinate)_2_ and unchelated ^52^Mn. Labelling conditions: § – 10^6^ cells incubated for 30 min at 37 °C in 3 mL cell medium; † – 5×10^6^ cells per mL incubated for 20 min at room temperature; ‡ – 10^8^ cells per mL incubated for 30 min at 37 °C. All values given as mean ± standard deviation. * – single experiment carried out in triplicate

Cell line labelled (LE = labelling efficiency)	[^52^Mn]Mn(oxinate)_2_ LE (%)	Unchelated ^52^Mn LE (%)
Breast cancer cells^§^ (MDA-MB 231)	33.4 ± 9.2 (*n* = 5)	3.9 ± 3.2 (*n* = 5)
Human embryonic kidney cells (HEK-293T)^§^	52.8 ± 3.8*	8.5 ± 0.6*
Gamma delta (γδ) T-cells^†^	45.6 ± 29.1 (*n* = 3)	0.9 ± 0.4 (*n* = 3)
Human blood platelets^‡^	14.8 ± 1.1*	2.7 ± 0.1*

These results together suggest that ^52^Mn leaves the cell *via* a specific cellular pathway. Manganese is a biologically essential metal found in a variety of tissues. It is particularly important for the catalytic activity of the mitochondrial enzyme superoxide dismutase (Mn-SOD) to detoxify reactive oxygen species.^21^,^22^ Mn is neurotoxic, and Mn^2+^ can cross the BBB barrier *via* the divalent metal transporter-1 (DMT-1).^23^–^25^ Hence, human biology has evolved mechanisms to manage and transport manganese ions efficiently, and calcium channels are known to be permeable to Mn^2+^. However, little is known about the intracellular handling of this element.^24^ Similar rapid cellular efflux was previously reported when labelling cells with ^64^Cu, using the lipophilic, redox-active complex Cu(PTSM) (PTSM = pyruvaldehyde-bis(N4-methylthiosemicarbazonate)) which is known to release copper bioreductively immediately on entry into cells.^26^,^27^ In these studies, after using this compound to label C6 glioma cells and murine Th1 cells, just 38% and 47%, respectively, of the radiolabel was retained 5 h post-labelling.^26^,^27^ Copper, like manganese, has several biological roles and the low retention of ^64^Cu may also be due to an active cellular process. If radiomanganese leaves the cell due to a cellular process, this may limit the technique of directly labelling cells using ^52^Mn ionophores. Although the tracer may be used to track cells at earlier time points, the rate of efflux means that the long half-life of the isotope – and the resulting radiation dose – cannot alone justify use of ^52^Mn for this application.

### Liposome (DOXIL/CAELYX) labelling with [^52^Mn]Mn(oxinate)_2_ for *in vivo* PET imaging

In addition of being used to label cells directly, our group has recently shown that oxine is an excellent ionophore for radiometal labelling of liposomal nanomedicines that encapsulate metal-chelating drugs, including preliminary *in vitro* work with ^52^Mn.^2^ Interestingly, [^52^Mn]Mn(oxinate)_2_ efficiently radiolabelled the FDA-approved nanomedicine DOXIL/CAELYX® (PEGylated liposomal doxorubicin) with high efficiency (>80%) and high *in vitro* human serum stabilities (*ca.* 95% after 72 h at 37 °C).^2^ The high retention of ^52^Mn inside the doxorubicin-loaded liposome is not surprising as Mn^2+^ has previously been shown to drive the loading of doxorubicin into liposomes by forming an stable intraliposomal Mn-doxorubicin complex.^28^ Abraham *et al.* characterised the complexation between Mn^2+^ and doxorubicin showing that the metal coordinates with a bidentate site consisting of the carbonyl and hydroxyl groups on the central aromatic ring system.^29^

The longitudinal imaging of liposomal medicines *in vivo* not only gives information on their biodistribution, but can be used to predict the efficacy of new treatments.^30^,^31^ In particular, imaging can provide information supporting ‘personalised medicine’ in which the response to liposomal therapies can be predicted from patient-to-patient. For this, particular focus has been placed on the use of nuclear imaging techniques because their high sensitivity gives them the ability to image therapeutic nanomedicines using very low doses which have a reduced or no physiological effect (microdosing).^32^ The radiolabelling technique developed by our group differs from previous methods developed in which the radiometal is chelated to ligands on a modified lipid bilayer,^30^,^33^,^34^ as well as direct incorporation of the radio-isotope into the lipid molecule itself.^35^ The use of radio-ionophore complexes for directly labelling liposomes with PET radionuclides has been previously reported by others with ^64^Cu (*t*_1/2_ = 12 h; *β*^+^ = 17.8%) and recently ^52^Mn – using a variety of ionophores, including oxine. However, in each case a drying step, involving high temperatures and a stream of argon is necessary to remove solvents before incubation with the liposomes.^36^,^37^ Additionally, the radiometal was trapped internally by an encapsulated DOTA chelator; hence modification of the liposomal medicine is necessary. By taking advantage of the chelating properties of some drugs, already-formulated liposomal nanomedicines can easily be labelled and tracked *in vivo* without the need for external modification of the nanomedicine or interfering with its manufacture – which may potentially affect the biodistribution of the nanodrug.

**Fig. 6:**
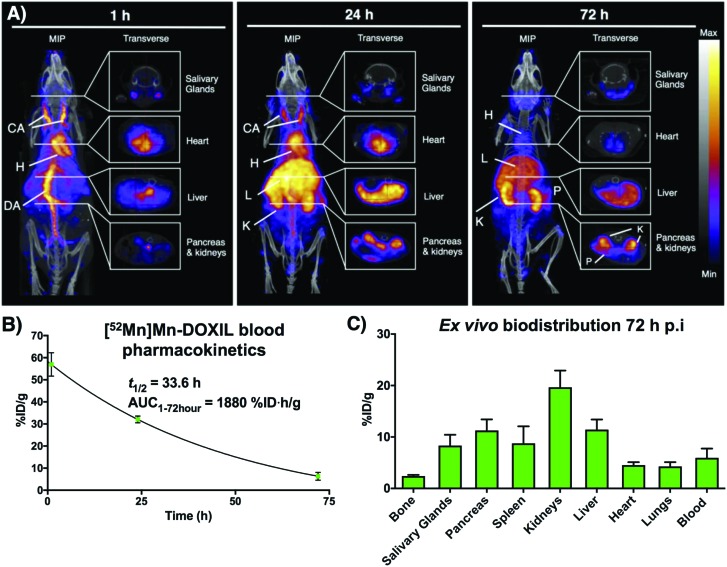
*In vivo* PET-CT imaging with [^52^Mn]Mn-DOXIL. (A) Maximum intensity projections and transverse slices through selected organs of PET/CT images of a B6CBAF1 mouse injected with [^52^Mn]Mn-DOXIL (1 MBq, 5 mg kg^–1^ doxorubicin dosage) at 1 h post-injection (p.i.), 24 h p.i. and 72 h p.i. CA = carotid arteries; H = heart; DA = descending aorta; L = liver; K = kidneys; S = spleen; SG = salivary glands; P = pancreas. (B) Blood pharmacokinetics of [^52^Mn]Mn-DOXIL in B6CBAF1 mice over 72 h and blood half-life of the liposomal nanomedicine. Error bars represent standard deviation (*n* = 3). (C) Radioactivity distribution of [^52^Mn]Mn-DOXIL at 72 h p.i. in B6CBAF1 mice. %ID g^–1^ were calculated from *ex vivo* gamma counting. Error bars represent standard deviation (*n* = 3).

Following on from our previous *in vitro* work describing the radiolabeling of DOXIL/CAELYX® ([^52^Mn]Mn-DOXIL) with ^52^Mn using oxine, here we describe the evaluation of the method to image the *in vivo* biodistribution and stability of [^52^Mn]Mn-DOXIL in mice using PET. Whilst previous work has described the labelling and imaging of DOXIL with technetium-99m,^38^ rhenium-186,^39^ and indium-111,^40^ this is the first time to the best of our knowledge that it has been tracked using PET imaging. [^52^Mn]Mn-DOXIL was prepared with a labelling efficiency of >80%. At 1 h post-injection (p.i.) of [^52^Mn]Mn-DOXIL (1 MBq, 5 mg kg^–1^ doxorubicin dose) in female B6CBAF1 mice, the majority of the radioactivity was observed in the bloodstream, with high signal in the heart and major blood vessels such as the carotid arteries and descending aorta (Fig. 6A). Some activity in these regions remained at 24 h p.i., with increasing uptake in the liver and spleen. This slow transition from the bloodstream at early time points to the gradual accumulation in the spleen and liver is typical of “stealth” PEGylated liposomal nanomedicines such as DOXIL; the stealth properties of these liposomes inhibit recognition by the mononuclear phagocyte system/reticuloendothelial system (MPS/RES).^41^,^42^ The circulation half-life of [^52^Mn]Mn-DOXIL was calculated by measuring radioactivity in blood samples taken at specific time points (Fig. 6B). The data were fitted to a one-compartment pharmacokinetic model that allowed the calculation of the circulation half-life (*t*_1/2_) and area under the curve (AUC). Thus, the calculated circulation half-life (*t*_1/2_) was 33.6 h, typical of a stealth nanomedicine and similar to that calculated by other preclinical studies with DOXIL/CAELYX (20–30 h).^38^,^39^,^42^ This also confirms that radiolabelling is stable while in the bloodstream (since the observed biodistribution does not match that of free ^52^Mn, see below).

Despite the confirmed stability of [^52^Mn]Mn-DOXIL in circulation and observation of the expected biodistribution at early time points, the PET images at 72 h p.i. (Fig. 6A) showed a profile characteristic of unchelated ^52^Mn (high signal in kidneys, pancreas and salivary glands^43^,^44^). This was confirmed by *ex vivo* biodistribution studies at 72 h p.i. (Fig. 6C) that demonstrated high radioactivity in these organs (kidneys (20 ± 3%ID g^–1^), pancreas (11 ± 2%ID g^–1^) and salivary glands (8 ± 2%ID g^–1^)). Conversely, spleen and liver uptake, commonly the organs with higher liposome presence at this timepoint, were relatively low (9 ± 3%ID g^–1^ and 11 ± 2%ID g^–1^, respectively) compared to other studies with stealth liposomes of similar physicochemical characteristics.^2^ Image-based quantification of the PET images is in agreement with these findings (Fig. S7†). Release of the contents of the liposomal nanodrug over time is expected, and indeed is required for anti-tumour efficacy, and has been previously observed. For example, previous work from our group noted increasing activity uptake in the bone over time when tracking ^89^Zr-labelled liposomal nanodrugs – indicative of the free radionuclide that is supposedly released when the liposomes extravasate and release their contents.^2^ Additionally, previous studies tracking DOXIL using [^99m^Tc]Tc- and [^186^Re]Re-BMEDA (*N*,*N*-bis(2-mercaptoethyl)-*N*′,*N*′-diethylethylenediamine) complexes reported increased kidney uptake after 40 h relating to release of the radioisotope from metabolised liposomes.^38^,^39^ However, efflux of the radionuclide from [^52^Mn]Mn-DOXIL occurs more rapidly and to a larger extent, with signs of radionuclide efflux at 24 h (high uptake in kidneys, Fig. 6A). We hypothesise this may be due to low cellular retention of the isotope within cells compared with ^89^Zr and the above-mentioned SPECT radionuclides, as our cell labelling results with [^52^Mn]Mn(oxinate)_2_ suggest (*vide supra*). Thus, after the accumulation and subsequent destruction of [^52^Mn]Mn-DOXIL within the liver, the isotope – along with the drug cargo – will be released. Consequently, ^52^Mn will return to the blood stream resulting in the free ^52^Mn biodistribution profile observed. Alternatively a similar process may occur after [^52^Mn]Mn-DOXIL is taken up by tissue/circulating macrophages. This may explain the lower-than-expected RES uptake observed at 72 h, compared to when tracking liposomes with ^89^Zr.^2^ Hence we propose that [^52^Mn]Mn(oxinate)_2_ may find applications in imaging liposomal biodistribution, and that the rapid efflux of released ^52^Mn from cells, and hence from tissues, makes the distribution to kidneys, pancreas and salivary glands a useful biomarker for the subsequent release of the drug cargo from liposomes *in vivo*.

## Conclusions

The simplicity, efficiency and effectiveness of 8-hydroxyquinoline (oxine) as an ionophore for the PET isotope ^52^Mn for cell/liposome radiolabelling has been demonstrated. We have provided supporting evidence that the most likely radiometal complex species that enter cells/liposomes is [^52^Mn]Mn(oxinate)_2_. In the context of cell labelling, [^52^Mn]Mn(oxinate)_2_ labels cells with comparable efficiency to [^89^Zr]Zr(oxinate)_4_, and does not cause cell death for up to 24 h at the activity levels used, but the technique is limited by rapid cellular efflux of ^52^Mn. Since manganese is an endogenous essential metal, the rapid efflux is likely to be due to a native cellular efflux pathway. As such, we suggest that direct cell labelling with [^52^Mn]Mn(oxinate)_2_ may be inappropriate for long-term *in vivo* cell tracking that fully exploits the long half-life of ^52^Mn. DOXIL labelling with [^52^Mn]Mn(oxinate)_2_ is highly efficient and DOXIL radiolabelled in this way has high radiochemical stability *in vivo* whilst in the bloodstream. After 24–72 h post injection, as the liposomes extravasate and enter tissues/cells, efflux of the radionuclide is evident from the characteristic high uptake of manganese in kidneys, pancreas and salivary glands, most likely reflecting lysis of liposomes and release of drug. Since these organs are not usually involved in liposomal nanomedicine clearance, the characteristic biodistribution of free ^52^Mn could be used as a marker of drug release from liposomes radiolabelled using this method. [^52^Mn]Mn(oxinate)_2_ may find other applications in the future as a method to incorporate tracer amounts of this essential metal inside cells to study its biological trafficking mechanisms *in vitro* and *in vivo*.

## Materials and methods

All chemical reagents were purchased from commercial sources. Water (18.2 MΩ cm) was obtained from an ELGA Purelab Option-Qsystem. Mass spectrometry (ES-TOF) analysis was conducted by Lisa Haigh of the Mass Spectrometry Service, Imperial College London. IR analysis was carried out using a PerkinElmer Spectrum 100 FT-IR spectrometer. UV titrations were carried out using a PerkinElmer Lambda 25 spectrometer, with samples in Brand 70 μL micro cuvettes. Electron paramagnetic resonance (EPR) spectroscopy was performed by Enrico Salvadori of the EPR Service, Queen Mary University London. DOXIL (Caelyx) was kindly provided by the Chemotherapy Department at Guys & St Thomas’ Hospital, London (UK). [^52^Mn]MnCl_2_ was produced by Jesper Fonslet from the Hevesy Lab, at the Technical University of Denmark. Radioactivity in samples were measured using CRC-25R dose calibrator (Capintec). iTLC-SG and SA strips were purchased from Agilent, UK and scanned using the PerkinElmer Cyclone Plus Storage Phosphor Imager. Gamma counting was performed using a Wallac 1282 CompuGamma γ counter. The human biological samples were sourced ethically and their research use was in accord with the terms of the informed consents under an Institutional Review Board/Ethics Committee (IRB/EC) approved protocol. All animal studies were ethically reviewed and carried out in accordance with the Animals (Scientific Procedures) Act 1986 and the GSK Policy on the Care, Welfare and Treatment of Animals.

### Synthesis of Mn(oxinate)_2_

An aliquot of 1 M KOH solution (690 μL, 0.69 mmol, 1 eq.) was added dropwise to a stirring mixture of 8-hydroxyquinoline (0.1 g, 0.69 mmol, 1 eq.) in ethanol (10 mL) (1 eq.) at 55 °C. The resulting solution was stirred at 55 °C for 0.5 h and then at room temperature (RT) for 2 h. Manganese(ii) chloride tetrahydrate (68 mg, 0.34 mmol, 0.5 eq.) in ethanol was then added dropwise and the mixture stirred for 2 h at RT. The resulting precipitate was then filtered and washed with ethanol and water, then dried *in vacuo* to leave a pale yellow solid (26 mg, 22%).

IR *ν*_max_ (cm^–1^): 3045 w (*ν*_CH_), 1605 m (*ν*_C=N_), 1572 vs (*ν*_C=C_), 1495 vs, 1461 vs, 1424 m, 1386 vs, 1375 vs, 1319 vs (*ν*_C–C_), 1279 (*ν*_C–N_) s, 1238 m, 1207 w, 1178 w, 1137 w, 1105 vs, 1059 m (*ν*_C–O_), 1034 m, 983 w, 960 w, 903 m, 870 m, 824 vs, 806 s, 787 vs, 746 vs, 739 vs, 652 m, 699 m, 583 m, 724 m.

HRMS (ES, +ve): calculated *m*/*z* for [M + H]^+^ 344.0352; found: 344.0348. Calculated *m*/*z* for (2[M] + H)^+^ = 687.0631; found: 687.0684. Calculated *m*/*z* for [M + H + C_2_H_3_N]^+^ = 385.0618; found: 385.0805*. Calculated *m*/*z* for [M + H + C_4_H_8_O_2_]^+^ = 432.0876; found: 432.1399*. * = *m*/*z* with mass error >10 ppm.

Elemental analysis: Calc. for C_18_H_12_MnN_2_O_2_: C, 53.82%; H, 3.01%; N, 6.97% Found: C, 53.68%; H, 3.88%; N, 6.99%.

### UV-titration of MnCl_2_ and 8-hydroxyquinoline

Within an ultra-micro cuvette a 0.1 mM solution of 8-hydroxyquinoline (1 mL, 1.46% v/v DMSO in 1 mM ammonium acetate solution pH 7–9) was prepared. The molar equivalents (0.1, 0.2, 0.3, 0.4, 0.5, 0.6, 0.8, 1, 2 and 3 eq.) of an aqueous manganese(ii) chloride tetrahydrate solution were then added sequentially, and the solution mixed with a 1000 μL pipette tip and left to stand for 2 min. Absorbance measurements were taken of the 0.1 mM oxine solution and after each addition of the manganese(ii) chloride solution. For the control titrations, manganese(ii) chloride were added to a solution containing no 8-hydroxyquinoline.

### EPR spectroscopy

A 1 mM solution of 8-hydroxyquinoline (1 mL, 1.46% v/v DMSO in 1 mM ammonium acetate solution pH 9) was prepared. 0.5 equivalents of an aqueous solution of manganese(ii) chloride tetrahydrate solution was added. EPR measurements of the resulting solution were performed at 100 K using an X/Q-band Bruker Elexsys E580 spectrometer (Bruker, Germany) equipped with a closed-cycle cryostat (Cryogenic Ltd, UK) controlled by a LakeShore temperature controller. Measurements were carried out in an X-band split-ring resonator module with 2 mm sample access (ER 4118X-MS2). Samples were loaded on Suprasil EPR tubes (Wilmad LabGlass) with OD = 1.6 mm, ID = 1.1 mm. Baseline spectra of samples containing only the buffer were also collected and used as a reference. All the spectra presented have been baseline-subtracted.

### [^52^Mn]MnCl_2_ production

^52^Mn was prepared according to the procedure described by Fonslet *et al.*^45^ In brief, the manganese was produced by 16 MeV proton irradiation of natural chromium. Separation of the ^52^Mn from the chromium target material was performed by four sequential anion exchange purifications, trapping the ^52^Mn out of ethanol–HCl mixtures.

### [^52^Mn]Mn(oxinate)_2_ radiosynthesis

An aliquot of [^52^Mn]MnCl_2_ was made up to 50 μL using chelex-treated water. 8-Hydroxyquinoline was dissolved in DMSO (12.5 mg mL^–1^) and an aliquot of this solution (4.8 μL) was added to the [^52^Mn]MnCl_2_ solution. The mixture was neutralised *via* addition of 0.1 M ammonium acetate (pH 7) and the mixture was then heated at 50 °C for 15 min. iTLC-SA conditions: *R*_f_ = 0.3–0.8, mobile phase = 20% MeOH in CHCl_3._ RCY % = 69 ± 20%.

For the preparation of the unchelated [^52^Mn]MnCl_2_ solution, the above procedure was carried out, however DMSO (4.8 μL) was added instead of the 8-hydroxyquinoline solution. iTLC-SA conditions: *R*_f_ = 0, mobile phase = 20% MeOH in CHCl_3_.

### MDA-MB 231/HEK-293T cell labelling with [^52^Mn]Mn(oxinate)_2_

Tissue culture 6-well plates were seeded with 8 × 10^5^ cells and left for 24 h in Dulbecco's Modified Eagle Medium (DMEM) with 10% fetal bovine serum (FBS). The medium was removed and replaced with 3 mL of serum-depleted medium (0% FBS). 50 kBq of [^52^Mn]Mn(oxinate)_2_ in serum-depleted medium (0% FBS) were added to the cells (0.05 Bq per cell) which were then incubated at 37 °C for 30 min. Subsequently, the cell medium was removed and the cells washed with PBS (2 mL) and trypsin (250 μL) was added and the cells incubated for 2 min to allow trypsinisation. Cell medium (750 μL) was then added and the cells re-suspended. The counts for the resuspended cells and the cell supernatant plus PBS washes were measured and the labelling efficiency calculated.

#### Cellular retention

The labelled cells were re-plated on a 6-well plate and cell medium (2 mL) was added. After incubation at 37 °C for 4 or 24 h, the cell medium was removed and the cells washed with PBS (2 mL) and trypsin (250 μL) was added and the cells incubated for 2 min to allow trypsinisation. Cell medium (750 μL) was then added and the cells re-suspended. The counts for the resuspended cells and the cell supernatant plus PBS washes were measured in a gamma-counter.

### Human blood platelet labelling with [^52^Mn]Mn(oxinate)_2_

A Sepharose 4B column was washed with ∼100 mL of HEPES Tyrode's buffer. 15 mL of donor blood were centrifuged at 120*g* for 10 min at 25 °C. The plasma layer (cloudy yellow top layer) was then removed and added to the column and eluted with HEPES Tyrode's buffer. The eluate was collected when it appeared cloudy (indicating the presence of platelets) and was subsequently centrifuged at 400*g* for 10 min and resuspended in PBS (6 mL). 500 μL aliquots were taken and labelled with 100 μL of the [^52^Mn]Mn(oxinate)_2_ and free ^52^Mn suspensions (in 1 mL serum-depleted medium) both in triplicate. The suspensions were incubated for 30 min at 37 °C, after which the suspensions were centrifuged at 120*g* for 10 min at 25 °C and the supernatant removed. The cell pellet and supernatant were then gamma counted.

### Gamma-delta T-cell labelling with [^52^Mn]Mn(oxinate)_2_

[^52^Mn]Mn(oxinate)_2_ and/or unchelated ^52^Mn (300 kBq) were added to 5 × 10^6^ γδ T-cells (0.06 Bq per cell) in suspension in sterile PBS (1 mL). The cells were incubated at room temperature with gentle mixing for 20 min. The cell suspensions were then centrifuged at 700*g* for 5 min. The supernatant was then removed and the cells were resuspended in PBS (500 μL) and centrifuged again at 700*g* for 5 min. The supernatants were combined and the cells resuspended in RPMI cell medium (1 mL). Aliquots (100 μL) of the cell suspension and the supernatant were then taken and gamma counted, and the labelling efficiency calculated.

#### Cellular retention

2.5 × 10^6^ of the labelled cells were then added to a 6-well cell culture plate and RPMI medium (3.5 mL) was then added. The cells were then incubated for 24 h at 37 °C. The cell suspensions were then centrifuged at 700*g* for 5 min. The supernatant was then removed and the cells were resuspended in PBS (500 μL) and centrifuged again at 700*g* for 5 min. The supernatants were combined and the cells re-suspended in RPMI cell medium (1 mL). Aliquots (100 μL) of the cell suspension and the supernatant were then taken and gamma counted, and the retention calculated.

### Cell supernatant log *P* measurements

0.5 MBq of [^52^Mn]Mn(oxinate)_2_ in serum-depleted medium (0% FBS) were added to 10^6^ cells on tissue culture plates in serum-depleted DMEM (3 mL) which were then incubated at 37 °C for 30 min. At each time point, 5 μL of the cell medium was removed and added to water/octanol (1 mL 1 : 1) and the mixture vortexed for 2 min, then centrifuged for 20 s. An aliquot (200 μL) from each layer was then removed to be gamma counted.

### Cell viability

After the labelling of the MDA-MB-231 cells with [^52^Mn]Mn(oxinate)_2_, the cell medium was removed and the cells washed with PBS (2 mL). The cell medium and PBS washes were combined in a 15 mL falcon and trypsin (250 μL) was added and the cells incubated for 2 min to allow trypsinisation. Cell medium (750 μL) was then added and the cells re-suspended and combined with cell medium and PBS washes, then centrifuged at 200*g* for 5 min. The supernatant was removed and the cell pellet resuspended in cell medium (1 mL). An aliquot (10 μL) was taken and mixed with trypan blue solution (10 μL). The number of dead and alive cells were then counted on a haemocytometer and the % cell viability calculated from the proportion of the two.

### DOXIL® labelling with [^52^Mn]Mn(oxinate)_2_

[^52^Mn]Mn(oxinate)_2_ (150 μL, 3.2% v/v DMSO in water) was added to a solution of DOXIL (350 μL, 2 mg mL^–1^) and the mixture heated with frequent agitation at 50 °C for 30 min. The mixture was then loaded onto a PD minitrap G-25 size exclusion column (GE Healthcare) following the manufacturers’ gravity protocol. The labelled liposomes ([^52^Mn]Mn-DOXIL) were then obtained by adding saline (750 μL) to the column and collecting the eluate.

### PET/CT imaging

Animal imaging studies were carried out in accordance with British Home Office regulations governing animal experimentation. Female B6CBAF1 mice (9 weeks old) were anesthetised with isofluorane (1.5–2%) during all imaging sessions. [^52^Mn]Mn-DOXIL (1 MBq, 150 μL, 5 mg kg^–1^ doxorubicin dosage) was injected *i.v.* into the mice (*n* = 3) at *t* = 0 h. PET/CT imaging was performed at *t* = 1 h, 24 h and 72 h (*n* = 1). PET/CT imaging was performed for 60 min on a nanoScan *in vivo* PET/CT preclinical imager (Mediso Medical Imaging Systems, Budapest, Hungary). All PET/CT data sets were reconstructed using a Monte Carlo based full 3D iterative algorithm (Tera-Tomo, Mediso Medical Imaging Systems, Budapest, Hungary). Decay correction to time of injection was applied. CT images were obtained with 55 kVp tube voltage, 1200 ms exposure time in 360 projections. All the images were analysed using VivoQuant software (inviCRO, USA).

### *Ex vivo* biodistribution

Biodistribution studies were carried out in accordance with British Home Office regulations governing animal experimentation. Three B6CBAF1 mice were culled by cervical dislocation whilst under anaesthesia, and the organs of interest were dissected, weighted and gamma-counted together with standard samples of the injected radiotracer to obtain percentages of the injected dose per mass values (%ID g^–1^) for each organ/tissue. Additionally, at *t* = 1, 24 and 72 h blood samples were taken. Whilst under anaesthetic, the tail vein of the mouse was pricked and blood taken up in a heparinised capillary tube (20 μL). Each sample was weighed and counted with a γ counter (LKB compugamma), together with standards prepared from a sample of the injected [^52^Mn]Mn-DOXIL.

## Author contributions

The manuscript was written through contributions of all authors. P. G. wrote the manuscript; P. G. and R. R. developed and performed the radiosynthesis and characterization; P. G. and F. M. performed the cell/platelet studies. J. F. produced and characterized ^52^Mn; J. B. and P. G. performed preclinical imaging studies; M. C., P. J. R., A. G. and P. J. B. provided supervision to P. G. in cell labeling (M. C., P. J. R, P. J. B.) and nanomedicine (A. G.); R. T. M. R. and N. L. conceived the study and supervised experiments, data analysis and the writing/editing of the manuscript with contributions from all authors.

## Conflicts of interest

There are no conflicts of interest to declare.

## Supplementary Material

Supplementary information
