# Supplementary material for: Manganese-52: applications in cell radiolabelling and liposomal nanomedicine PET imaging using oxine (8-hydroxyquinoline) as an ionophore
Source: Dalton Trans. 2018 May 24;47(28):9283–93. doi: 10.1039/c8dt00100f (PMC6049564; doi:10.1039/c8dt00100f)
Supplement: Supplementary file 1 [file DT-047-C8DT00100F-s001.pdf]

Electronic Supplementary Material (ESI) for Dalton Transactions.
This journal is © The Royal Society of Chemistry 2018

Electronic Supplementary Information (ESI)

Manganese-52: applications in cell radiolabelling and liposomal nanomedicine PET imaging using oxine (8-hydroxyquinoline) as an ionophore.

Peter Gawne,[a] Francis Man,[a] Jesper Fonslet,[b] Riya Radia,[a] Jayanta Bordoloi,[a] Matthew Cleveland,[c] Pilar Jimenez-Royo,[c] Alberto Gabizon,[d] Philip J. Blower,[a] Nicholas Long[e] and Rafael T. M. de Rosales*[a]

[a] School of Biomedical Engineering & Imaging Sciences, King's College London, St. Thomas' Hospital, London, SE1 7EH, U.K.
[b] The Hevesy Lab, Technical University of Denmark, 4000 Roskilde, Denmark.
[c] GSK Medicines Research Centre, Gunnels Wood Road, Stevenage, Hertfordshire, SG1 2NY, U.K.
[d] Oncology Institute, Shaare Zedek Medical Center and Hebrew University−School of Medicine, Jerusalem 9103102, Israel.
[e] Department of Chemistry, Imperial College London, South Kensington Campus, London SW7 2AZ, U.K.

E-mail: rafael.torres@kcl.ac.uk

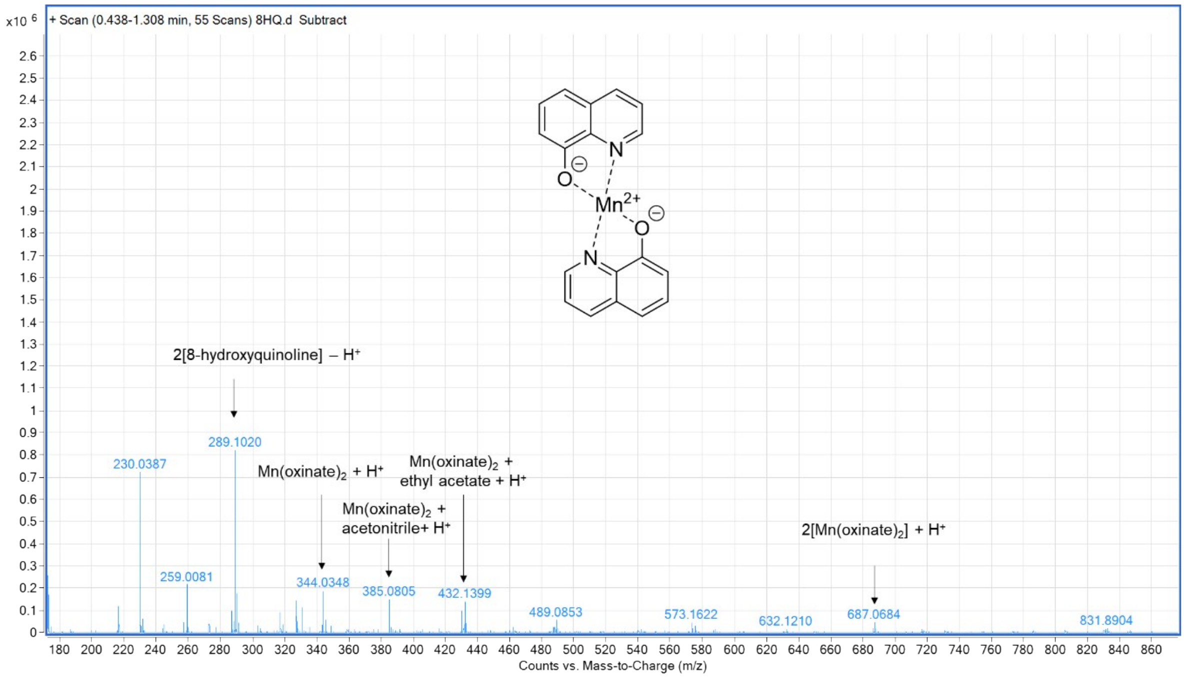

Figure S1: Mass spectrum (ESI, +ve mode) of Mn(oxinate)$_2$. Mass peak at 343.0348 corresponds to the bis-oxine manganese(II) complex $[M + H]^+$.

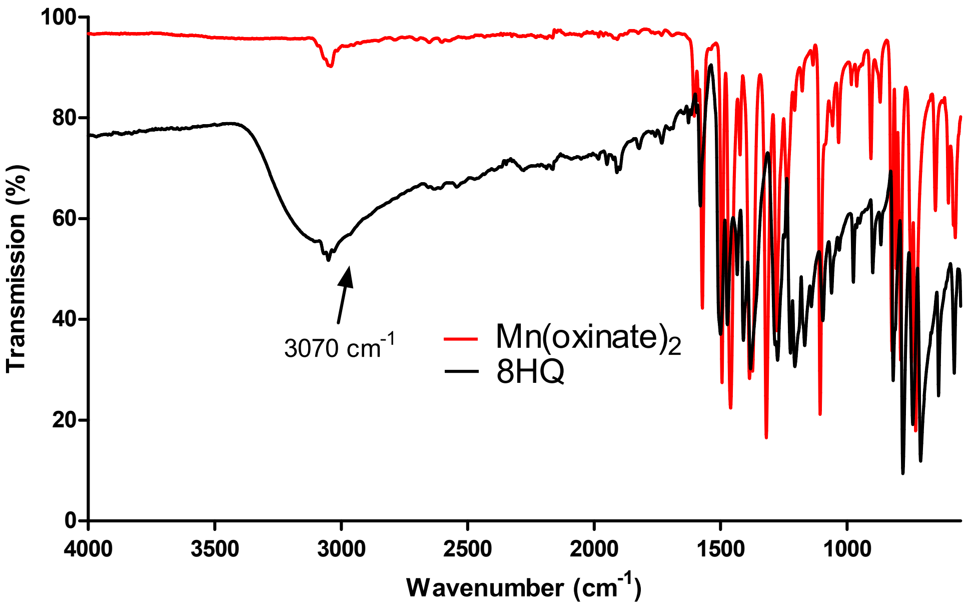

Figure S2: Infra-red spectrum of 8-hydroxyquinoline (black line) and Mn(oxinate)$_2$ (red line). After reaction with $MnCl_2$, a reduction of the broad O-H stretch band at 2700-3400 cm^{-1} occurs - relating to the formation of the Mn-O bond.

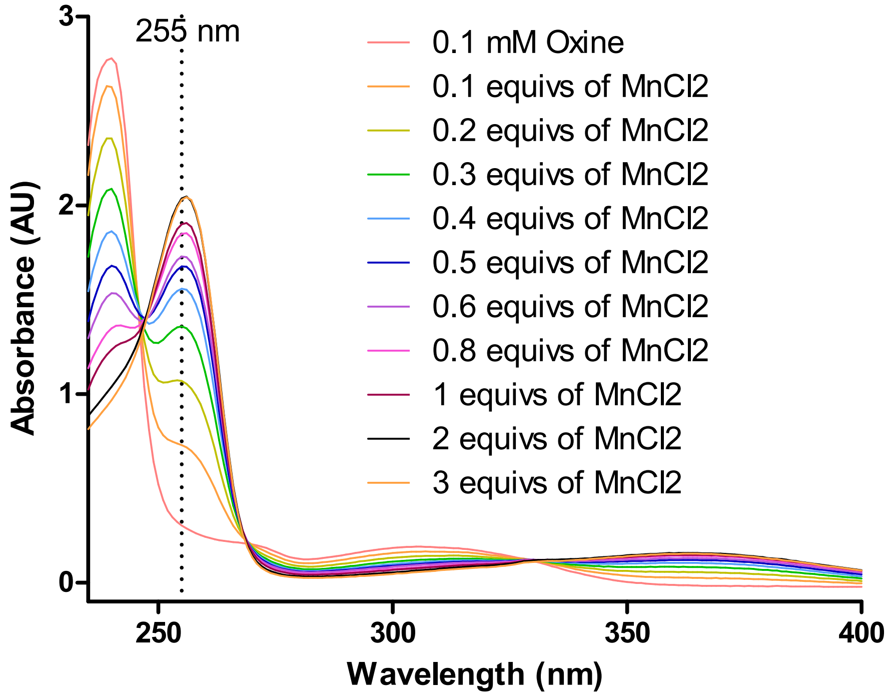

Figure S3: Representative UV-vis titration of $MnCl_2$ into a 0.1 mM solution of oxine at pH 9.

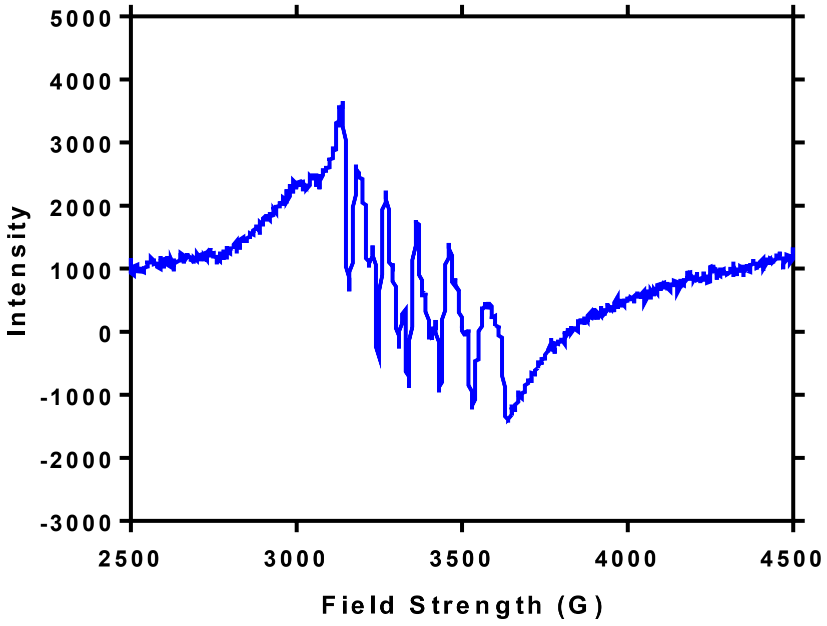

Figure S4: EPR spectrum of a 1 mM oxine solution (in 1 mM ammonium acetate buffer, pH 9) after addition of 0.5 equivalents of $MnCl_2$ at 100 K. Formation of a Mn^{2+} species is clearly evident.

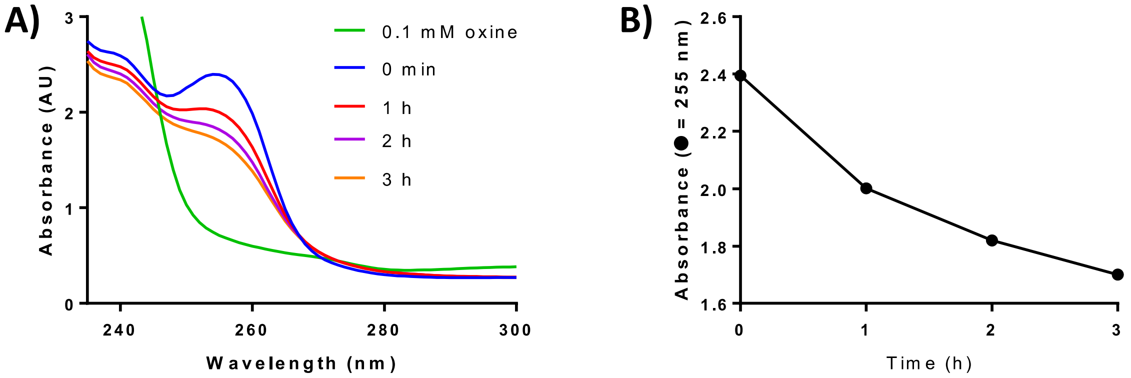

Figure S5: **A)** Overlaid UV-vis spectra of a 0.1 mM oxine solution (in 1 mM ammonium acetate buffer, pH 9, green line) and at different time points after addition of 0.5 equivalents of $MnCl_2$; **B)** plot of absorbance at λ = 255 nm versus hours after addition of $MnCl_2$.

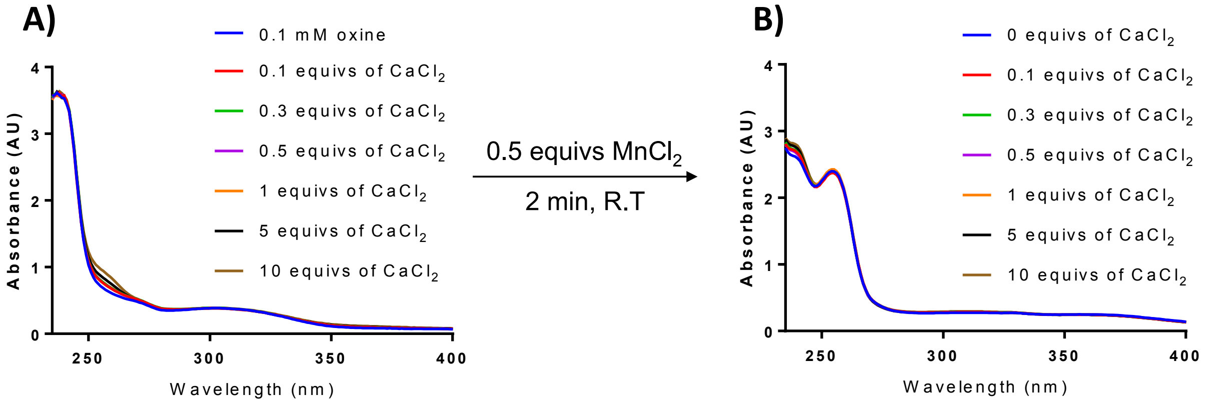

Figure S6: A) Overlaid UV-vis spectra of a 0.1 mM oxine solution in 1 mM ammonium acetate buffer (pH 9) in the absence and presence of various equivalents of $CaCl_2$; **B)** Overlaid UV-vis spectra after addition of 0.5 equivalents of $MnCl_2$ to each of these solutions. The presence of calcium chloride appeared to have no inhibitory effect on the $Mn(oxinate)_2$ complex being formed *in situ.*

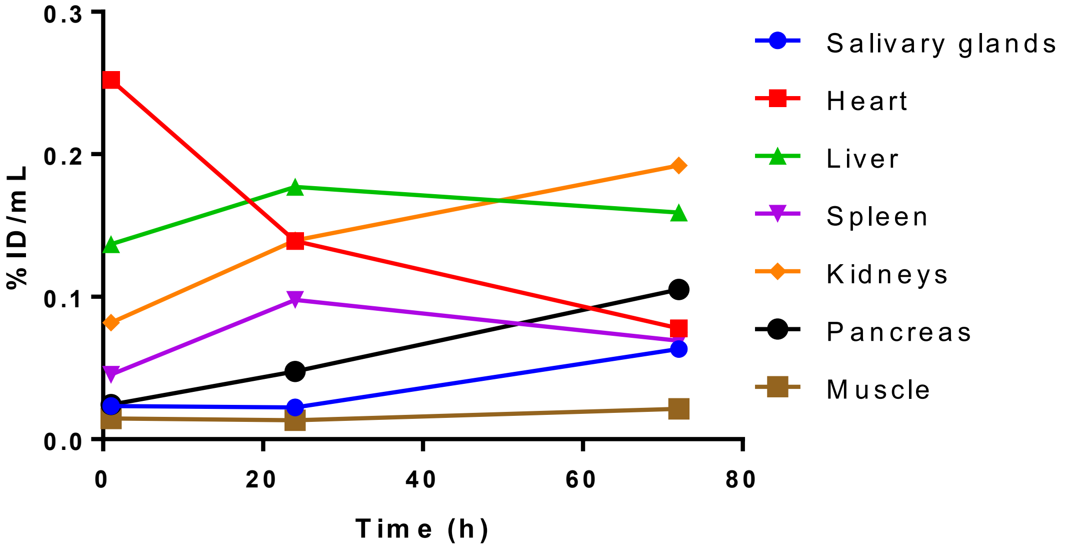

Figure S7: Time-activity curve based on the imaging study shown in Figure 6 (n = 1). A gradual clear increase in activity in the pancreas, salivary glands and kidneys is consistent with the release of ^{52}Mn from the [^{52}Mn]Mn-DOXIL complex.
